# Catatonia phenomenology and treatment response in inpatient psychiatry units in Oman

**DOI:** 10.1192/j.eurpsy.2026.11539

**Published:** 2026-07-31

**Authors:** M. M. M. Al-Zadjali

**Affiliations:** Psychiatry, MCMSS, Muscat, Oman

## Abstract

**Introduction:**

Catatonia is a neuropsychiatric syndrome that is hardly understood and difficult to diagnose leading to a delay in the diagnosis and thus the treatment. It can be challenging to identify, especially with patients that suffer from other medical or neurological co-morbidities and therefore 59% of those patients are underdiagnosed. Its incidence among acute psychiatric illnesses varies between 6% to 38% with a prevalence of 10% to 15% among acute psychiatric inpatients.

**Objectives:**

- Study the clinical manifestations of catatonia in Oman.
- Study the types of treatment of catatonia in Oman.
- Investigate the patients’ response to each kind of treatment.

**Methods:**

A retrospective observational study that was conducted in two tertiary hospitals in Oman; Al Masarah Hospital and Sultan Qaboos University Hospital. Inclusion criteria had all inpatients above 18 years old who were diagnosed with catatonia according to Diagnostic Statistical Manual V during the period of 2018 to 2022. Patients younger than 18 years old and patients who left against medical advice were excluded from this study. Bush-Francis Catatonia Rating Scale was also used to asses the presence and severity of the catatonia.

**Results:**

Our study included 49 inpatients with a mean age of 28.1 years (range:18-57) with females (n=27) than males (n=22). According to BRCRS, mutism (73.5%), withdrawal (69.4%), posturing/catalepsy (67.3%), negativism (67.3%) and excitement (49.0%) were the most common catatonic symptoms observed. The most commonly recorded diagnosis was catatonia associated with schizophrenia (36.7%) followed by catatonia associated with major depressive disorder (22.4%). The severity score had a mean of 12.3 (median=11, mode=11) with a mean of 4.8 in screening score (median=5, mode=5). The mean duration of illness was 23 days (range:2-250) with an average of 29 days (range 1-135) of admission. Majority of the patients (79.6%) were given benzodiazepines as first line treatment and two thirds (67.3%) reported with an overall improvement. ECT sessions were also done on 6 patients with an overall improvement (83.3%).

**Conclusions:**

Our findings were aligned with those found in the literature. The most common diagnosis was catatonia due to schizophrenia followed by catatonia due to major depressive disorder with more males affected than females. Mutism, withdrawal, posturing and catalepsy were of the most common signs and symptoms seen in these catatonic inpatients. Treatment using benzodiazepines relieved two thirds of the patients. Moreover, ECT sessions has proven its efficacy in the improvement of the catatonic patients’ condition. Our findings provide further evidence to support previous literature regarding catatonia in terms of disease presentation, treatment modalities and patient response.

**Disclosure of Interest:**

None Declared

